# Characteristics of Australian cohort study participants who do and do not take up an additional invitation to join a long-term biobank: The 45 and Up Study

**DOI:** 10.1186/1756-0500-5-655

**Published:** 2012-11-27

**Authors:** Emily Banks, Nicol Herbert, Tanya Mather, Kris Rogers, Louisa Jorm

**Affiliations:** 1 National Centre for Epidemiology and Population Health, Australian National University, Canberra, Australia; 2 The Sax Institute, Sydney, Australia; 3 School of Medicine, University of Western Sydney, Sydney, Australia

## Abstract

**Background:**

Large-scale population biobanks are critical for future research integrating epidemiology, genetic, biomarker and other factors. Little is known about the factors influencing participation in biobanks. This study compares the characteristics of biobank participants with those of non-participants, among members of an existing cohort study.

**Methods:**

Individuals aged 45 and over participating in The 45 and Up Study and living ≤20km from central Wagga Wagga, New South Wales (NSW), Australia (rural/regional area) or ≤10km from central Parramatta, NSW (urban area) (n=2340) were invited to join a biobank, giving a blood sample and having additional measures taken, including height, weight, waist circumference, heart rate and blood pressure.

**Results:**

The overall uptake of the invitation to participate was 33% (762/2340). The response rate was 41% (410/1002) among participants resident in the regional area, and 26% (352/1338) among those resident in the urban area. Characteristics associated with significantly decreased participation were being aged 80 and over versus being aged 45–64 (participation rate ratio: RR = 0.45, 95%CI 0.34-0.60), not being born in Australia versus being born in Australia (0.69, 0.59-0.81), having versus not having a major disability (0.54, 0.38-0.76), having full-time caregiving responsibilities versus not being a full-time carer (0.62, 0.42-0.93) and being a current smoker versus never having smoked (0.66, 0.50-0.89). Factors associated with increased participation were being in part-time work versus not being in paid work (1.24, 1.07-1.44) and having an annual household income of ≥$50,000 versus <$20,000 (1.50, 1.26-1.80).

**Conclusions:**

A range of socio-economic, health and lifestyle factors are associated with biobank participation among members of an existing cohort study, with factors relating to health-seeking behaviours and access difficulties or time limitations being particularly important. If more widespread participation in biobanking is desired, particularly to ensure sufficient numbers among those most affected by these issues, specific efforts may be required to increase participation in certain groups such as migrants, the elderly, and those in poor health. Whilst caution should be exercised when generalising estimates of absolute prevalence from biobanks, estimates for many internal comparisons are likely to remain valid.

## Background

In recent years, researchers have sought to integrate genetic and other biomarker data with epidemiological data by obtaining biospecimens from participants in large-scale cohort studies. However, participation rates in biobank studies vary considerably [1-8] and are generally lower than for non-biomarker oriented research [2]. The limited evidence available suggests that older individuals, ethnic minorities, and those without a family history of illness are less likely to donate to biobanks than other potential participants [3,5,6]. Despite its implications for prevalence estimates and other aspects of biobank metholodogy, how participation relates to broader demographic, health and lifestyle factors is not known. This study investigates the relation of demographic, lifestyle and other personal characteristics, to participation in a biobank among members of an existing cohort study.

## Methods

### Overview

The 45 and Up Study is a large scale study of healthy ageing involving men and women aged 45 years and over from the general population of New South Wales (NSW), Australia and is described in detail elsewhere [9]. Briefly, from February 2006 to April 2009, men and women from the general population of NSW were sampled through the Medicare Australia database and joined the study by completing a postal questionnaire. They gave written informed consent for follow-up through repeated contact and linkage to population health databases. In keeping with many contemporary cohort studies with extensive linkage, the response rate for the 45 and Up Study as a whole is estimated to be 18% [9], with younger individuals, those living in major cities and men being underrepresented, compared to the general population [10]. Compared to a more representative population sample, cohort members were on average wealthier, less likely to smoke and more likely to have good, very good or excellent self-reported health [10].

The specific study described in this paper is known as the “Link-Up Study” and was conducted among a subset of 2340 individuals already taking part in The 45 and Up Study. Its primary aim was to compare response rates to an invitation to participate in a biobank according to different types of recruitment site, fasting status and reminder letter at an urban and regional location, using a randomised design. The results of the primary randomised analyses are reported elsewhere [11]; this paper focuses on the comparison of characteristics of those who did and did not take up the invitation to participate in the biobank.

### Procedure

All 45 and Up Study participants who resided ≤20km from central Wagga Wagga, NSW, Australia (a rural/regional area) or ≤10km from central Parramatta, NSW (an urban area) were invited to participate in this sub-study. Eligible 45 and Up Study cohort members were sent a postal invitation to take part in the Link-Up Study, which included a covering letter from the 45 and Up Study, a participant information leaflet and a brief questionnaire. The consent form was on the reverse of the questionnaire. Participants gave consent for the collection, long-term storage and use of their blood sample for unspecified health research, including genetic research. Participants gave their consent on the understanding that they would not receive any direct results from tests on their blood sample. In addition, participants agreed to their questionnaire answers and biomarker data being combined with existing data from the 45 and Up Study, including the baseline questionnaire and linkage to various population health databases.

#### Self-administered questionnaire

Participants were instructed to complete the questionnaire prior to attending their designated collection site and return it when they attended to give a blood sample. The questionnaire was one A4 page, and repeated selected questions from The 45 and Up Study baseline questionnaire. Questions included the participant’s date of birth, self reported height and weight, self-rated health, health events in the last 3 years, and the Medical Outcomes Score-Physical Functioning scale (MOS-PF). The data from this questionnaire were not used further in these analyses.

#### Biospecimen collection

Participants were randomised to donate biospecimens at either a dedicated clinic established specifically for the purposes of the Link-Up Study, or to attend at an existing local pathology centre (see [11] for details of this aspect of the study). The phlebotomists at each site took a 30ml blood sample from participants and measured height, weight, waist circumference, blood pressure and heart rate.

## 45 and Up Study baseline questionnaire

Information from the 45 and Up Study baseline questionnaire was used as the source of data to compare the characteristics of participants and non-participants in the Link-Up biobank and included: age; sex; education; country of origin (born in Australia/not born in Australia); whether a participant was caring for a sick or disabled family member or friend; self-report of assistance required with day-to-day tasks due to illness or disability; smoking status; body mass index; and physical limitations. Physical limitations were measured using the MOS-PF, which asks participants whether they are limited in their ability to perform vigorous and moderate physical activities and tasks such as: lifting shopping; climbing stairs; walking; bending, kneeling or stooping; and bathing or dressing [12]. This score was categorised into 4 groups: 100 (no physical limitations reported), 90–99, 60–89, and 0–59. Body mass index was calculated as the reported weight in kilograms divided by the reported height in metres, squared, and was classified according to World Health Organization criteria into underweight (<18.5kgm^-2^), normal weight (18.5-24.9kgm^-2^), overweight (25.0-29.9kgm^-2^) and obese (≥30.0kgm^-2^) [13].

### Outcome measures

The primary outcome measure for this study was participation in the Link-Up Study, through attendance at a biospecimen collection site to provide a blood sample and giving written consent to participate in the biobank, including use of blood samples for genetic and other research. The proportion of invitees who went on to participate in this way in the Link-Up Study is termed the “response rate”.

### Statistical methods

Response rate was analysed using generalised linear model with a binomial distribution and a log link function (binomial regression) (proc genmod in SAS, v9.2 Cary NC, USA) with response (yes or no) as the outcome. Associations with exposure variables were expressed as a participation rate ratio. This model was used to examine which individual characteristics were associated with differences in response rates. This model was not initially stratified by area. Model fit was assessed using the deviance to compare the fitted model with the full or saturated model that provided a perfect fit to the data. Wald 95% confidence intervals were calculated for rate ratio estimates, and the overall effect of a variable was assessed with a likelihood ratio test. Heterogeneity of the effect of personal variables on response rate between areas was assessed by sequentially adding an interaction term of area with each variable, and the effect assessed by a likelihood ratio test of the interaction.

### Ethics statement

Ethical approval for the study was provided by the University of New South Wales, Human Research Ethics Committee, the Australian National University Human Research Ethics Committee, and the University of Western Sydney Human Research Ethics Committee.

## Results

### Overall response to the invitation to participate

The overall uptake of the invitation to participate in the Link-Up Study was 33% (762/2340). The response rate was 41% (410/1002) among participants resident in the regional area (Wagga Wagga) and 26% (352/1338) among those resident in the urban area (Parramatta)(p<0.0001).

### Response rate according to demographic, lifestyle and other factors

Table 1 shows the response rate to the invitation to take part in the Link-Up Study among people already participating in the 45 and Up Study, according to a range of factors. No significant variation in response rate was observed according to sex, distance from collection site, time since joining the baseline 45 and Up Study and body mass index. Individuals aged 80 and over were significantly less likely to take part than younger individuals, as were those who were not born in Australia, compared to Australian-born individuals. People with a higher annual household income and those in part-time work were significantly more likely than others to take part. People who were caring full-time for a sick or disabled family member or friend were significantly less likely to take part in the Link-Up Study than people who did not have such caring responsibilities. Individuals needing help with day-to-day tasks because of a long-term disability or illness and those with a severe functional limitation due to their physical health were also significantly less likely to participate, compared to those without such disability. Compared to people who had never smoked, current smokers were less likely to take part. In the regional area, people with a tertiary education were significantly more likely to participate than those without a tertiary education; an attenuated relationship was observed in the urban area (p(interaction)=0.049). None of the relationships with the other factors varied significantly between the urban and regional areas (data not shown).

**Table 1:** Response rate to the invitation to take part in the Link-Up Study, according to demographic, lifestyle and other factors

**Characteristic**	**Total**	**Participated**	**Rate Ratio (crude) of participation**
	**N**	**N (%)**	**(95% CI)**
**Distance of residence from collection centre**			
<5km	1170	387 (33%)	1.00
≥5km	1170	375 (32%)	0.97 (0.87 - 1.09)
**Time since joining The 45 and Up Study**			
Less than 1 year	1128	381 (34%)	1.00
1 - <2 years	855	278 (33%)	0.98 (0.86 - 1.11)
2 years or more	357	103 (29%)	0.87 (0.73 - 1.04)
**Age**			
45-64 yrs	1464	499 (34%)	1.00
65-79 yrs	611	223 (36%)	1.07 (0.95 - 1.21)
≥80 yrs	265	40 (15%)	0.45 (0.34 - 0.60)
**Sex**			
Male	1107	348 (31%)	1.00
Female	1233	414 (34%)	0.94 (0.84 - 1.06)
**Educational qualifications**			
Wagga Wagga			
Not Tertiary Educated	765	287 (38%)	1.00
Tertiary Educated	221	118 (53%)	1.37 (1.18 - 1.60)
Parramatta			
Not Tertiary Educated	971	244 (25%)	1.00
Tertiary Educated	336	102 (31%)	1.20 (0.99 - 1.46)
**Country of origin**			
Born in Australia	1696	608 (36%)	1.00
Not born in Australia	644	154 (23%)	0.69 (0.59 - 0.81)
**Annual household pre-tax income**			
<$20,000	444	106 (24%)	1.00
$20,000-$49,999	535	174 (33%)	1.37 (1.12 - 1.68)
≥$50,000	1249	455 (36%)	1.50 (1.26 - 1.80)
**Employment status**			
Not in paid work	1021	309 (30%)	1.00
Part time paid work	433	160 (37%)	1.24 (1.07 - 1.44)
Full time paid work	837	282 (34%)	1.10 (0.97 - 1.25)
**Carer status**			
Not a full-time carer	2242	743 (33%)	1.00
Full-time carer	94	19 (20%)	0.62 (0.42 - 0.93)
**Disability**			
No major disability	2092	714 (31%)	1.00
Major disability	137	26 (18%)	0.54 (0.38 - 0.76)
**Physical functional limitations**			
None	719	242 (34%)	1.00
Mild	591	245 (41%)	1.22 (1.06 - 1.39)
Moderate	450	152 (34%)	1.00 (0.85 - 1.18)
Severe	352	72 (20%)	0.61 (0.49 - 0.77)
**Smoking status**			
Never smoker	1425	497 (35%)	1.00
Current smoker	159	37 (23%)	0.66 (0.50 - 0.89)
Ex-smoker	736	225 (31%)	0.88 (0.78 - 1.00)
**Body mass index**			
Underweight	38	9 (24%)	0.95 (0.80 - 1.12)
Normal weight	702	227 (32%)	1.00
Overweight	838	292 (35%)	1.08 (0.94 - 1.24)
Obese	516	158 (31%)	0.73 (0.41 - 1.31)

Note: Numbers do not always add up to total due to missing values.

## Discussion

Among individuals already taking part in a cohort study, people who participated in an biobank, through donating a blood sample and having physical measurements taken, were, on average, significantly younger, wealthier, more educated, less disabled and more likely to be born in Australia, compared to cohort members who did not participate. Those in part-time work were more likely to participate, and smokers and those with caregiving responsibilities were less likely to participate than other cohort members. No significant variation in response rate was observed according to sex, distance from collection site, time since joining the baseline 45 and Up Study and body mass index.

Research has previously been undertaken to investigate whether individuals would be willing to participate in biobanking, using hypothetical scenarios [14-17]. Past studies indicate that individuals may be reluctant to donate because of lack of personal relevance, concerns that their sample will be misused, and general distrust of biobanks or researchers [1,18]. However, data comparing the characteristics of individuals who actually do or do not participate in biobanks are limited. Consistent with results from this study, previous studies have found that ethnic minorities and older people are less likely than others to donate biospecimens for research [19-21]. We were unable to identify any studies that evaluated directly the influence of health and lifestyle characteristics on biobank participation; the factors we identified are similar to those that influence participation in research in general (e.g. “the healthy cohort effect”) [22-24]. Overall, our findings suggest that individuals less engaged in health-seeking behaviours (e.g. smokers) were less likely to participate, as well as those potentially affected by access difficulties or time limitations, such as those with disabilities or carer responsibilities, or the elderly.

Participation rates and factors influencing participation in biobanks vary from study to study, and according to context [1-4,8]. Even with the immense resources of the UK Biobank, a response rate of <10% was achieved. It has been suggested that those donating to biobanks should be representative of the general public [3]. Although this is generally seen as important for reliable estimates of point prevalence, experience to date indicates that this is unlikely to be practical on a large scale. Furthermore, participants do not need to be representative of the general population to make internal comparisons of relative risk within the cohort (i.e. to quantify associations between exposures and outcomes), which is generally the central purpose of large scale, long term biobanks. Such estimates of relative risk remain valid, even when the cohort is from a relatively select group [10,25]. Examples of highly selected, yet valid, internal comparisons include estimates of the relative risks of lung cancer for smokers versus non smokers, among British Doctors [26], and consistent estimates of breast cancer risk in users versus non users of the oral contraceptive pill, in a variety of cohort studies [27]. For this study, this means that although the absolute response rates to the invitation may not necessarily be generalisable to other studies, internal comparisons, including characteristics of responders versus non-responders, are likely to be both valid and generalisable [10,25]. More importantly, the implication for biobanks generally are that response rates and the characteristics of responders and non-responders may influence the generalisability of prevalence estimates from biobanks, but are less likely to be a problem for internal comparisons.

Another important consideration for long term biobanks is the need to include sufficient numbers of participants from specific population groups of interest to allow appropriately powered analyses. Our results indicate that certain groups are less likely to participate and strategies to enhance participation in these groups may be necessary to ensure appropriate heterogeneity in the biobank population. Such strategies could include targeted community-based recruitment of the elderly and migrant groups and home-based recruitment methods.

## Conclusions

People participating in research studies involving biobanking differ from those who do not take part in a number of ways, and are generally in better health. In keeping with cohort studies in general, this means that caution should be exercised in generalising from estimates of absolute prevalence from biobank studies, however for many internal comparisons, estimates are likely to remain valid.

## Abbreviations

CI: Confidence interval; MOS-PF: Medical Outcomes Score-Physical Functioning.

## Competing interests

The authors declare that they have no competing interests.

## Authors' contributions

Study conception and design: EB, LJ. Study coordination and data collection: NH, EB. Biostatistical supervision and conduct of analyses: KR. Interpretation of analyses: EB, LJ, KR. Literature review: TM, NH. Drafting of manuscript: EB, NH, TM. Revision of manuscript for important intellectual content: NH, KR, TM, LJ. All authors read and approved the final manuscript.

## References

[B1] Melas PA Sjöholm LK Forsner T Edhborg M Juth N Forsell Y Lavebratt C Examining the public refusal to consent to DNA biobanking: empirical data from a Swedish population-based study J Med Ethics 2010 36 2 93 98 10.1136/jme.2009.032367 20133403

[B2] Matsui K Kita Y Ueshima H Informed consent, participation in, and withdrawal from a population based cohort study involving genetic analysis J Med Ethics 2005 31 7 385 392 10.1136/jme.2004.009530 15994356 PMC1734191

[B3] Lanfear DE Jones PG Cresci S Tang F Rathore SS Spertus JA Factors influencing patient willingness to participate in genetic research after a myocardial infection Genome Med 2011 3 6 1 8 21676259 10.1186/gm255 PMC3218813

[B4] Levy D Splansky GL Strand NK Atwood LD Benjamin EJ Blease S Cupples LA D'Agostino RB Fox CS Kelly-Hayes M Consent for genetic research in the Framingham heart study Am J Med Genet A 2010 152A 5 1250 1256 10.1002/ajmg.a.33377 20425830 PMC2923558

[B5] Ford BM Evans JS Stoffel EM Balmaña J Regan MM Syngal S Factors associated with enrollment in cancer genetics research Canc Epidemiol Biomarkers Prev 2006 15 7 1355 1359 10.1158/1055-9965.EPI-05-0816 16835336

[B6] Bogner HR Wittink MN Merz JF Straton JB Cronholm PF Rabins PV Gallo JJ Personal characteristics of older primary care patients who provide a buccal swab for apolipoprotein E testing and banking of genetic material: the spectrum study Publ Health Genom 2004 7 4 202 210 10.1159/000082263 PMC2804857 15692195

[B7] Meschia JF Merino JG Willingness of ischemic stroke patients to donate DNA for genetic research: a systematic review J Stroke Cerebrovasc Dis 2003 12 5 228 231 10.1016/j.jstrokecerebrovasdis.2003.09.004 17903932

[B8] UK Biobank Coordinating Centre UK biobank: report of the integrated pilot phase 2006 Cheshire http://www.ukbiobank.ac.uk/wp-content/uploads/2011/06/Pilot_report.pdf?phpMyAdmin=trmKQlYdjjnQIgJ%2CfAzikMhEnx6

[B9] Banks E Redman S Jorm L Armstrong B Bauman A Beard J Beral V Byles J Corbett S Cumming R Cohort profile: the 45 and Up study Int J Epidemiol 2008 37 5 941 947 17881411 10.1093/ije/dym184 PMC2557061

[B10] Mealing NM Banks E Jorm LR Steel DG Clements MS Rogers KD Investigation of relative risk estimates from studies of the same population with contrasting response rates and designs BMC Med Res Methodol 2010 10 26 10.1186/1471-2288-10-26 20356408 PMC2868856

[B11] Banks E Herbert N Rogers K Mather T Jorm L Randomised trial investigating the relationship of response rate for blood sample donation to site of biospecimen collection, fasting status and reminder letter: the 45 and Up study BMC Med Res Methodol 2012 12 147 10.1186/1471-2288-12-147 23006657 PMC3532153

[B12] Hays RD Liu H Spritzer K Cella D Item response theory analyses of physical functioning items in the medical outcomes study Med Care 2007 45 5 S32 S38 17443117 10.1097/01.mlr.0000246649.43232.82

[B13] World Health Organization Obesity: preventing and managing the global epidemic 1998 Geneva: World Health Organization 11234459

[B14] Kettis-Lindblad A Ring L Viberth E Hansson MG Genetic research and donation of tissue samples to biobanks. What do potential sample donors in the Swedish general public think? Eur J Publ Health 2006 16 4 433 440 10.1093/eurpub/cki198 16207726

[B15] Goddard KAB Smith S Chen C McMullen C Johnson C Biobank recruitment: motivations for nonparticipation Biopreservation Biobanking 2009 7 2 119 121 10.1089/bio.2009.0006 PMC3205734 22087353

[B16] Hoeyer K Olofsson B-O Mjörndal T Lynöe N Informed consent and biobanks: a population-based study of attitudes towards tissue donation for genetic research Scand J Publ Health 2004 32 3 224 229 10.1080/14034940310019506 15204184

[B17] Wong ML Chia KS Yam WM Teodoro GR Lau KW Willingness to donate blood samples for genetic research: a survey from a community in Singapore Clin Genet 2004 65 1 45 51 15032974 10.1111/j..2004.00192.x

[B18] Bussey-Jones J Garrett J Henderson G Moloney M Blumenthal C Corbie-Smith C The role of race and trust in tissue/blood donation for genetic research Genet Med 2010 12 2 116 121 10.1097/GIM.0b013e3181cd6689 20098329 PMC3114600

[B19] Sanner JE Frazier L Factors that influence characteristics of genetic biobanks J Nurs Scholarsh 2007 39 1 25 29 10.1111/j.1547-5069.2007.00139.x 17393962

[B20] Neumark DE Stommel M Given CW Given BA Research design and subject characteristics predicting nonparticipation in a panel survey of older families with cancer Nurs Res 2001 50 6 363 368 10.1097/00006199-200111000-00006 11725938

[B21] McQuillan GM Porter KS Consent for future genetic research: the NHANES experience in 2007–2008 IRB 2011 33 1 9 14 21314035

[B22] Goldberg M Chastang JF Leclerc A Zins M Bonenfant S Bugel I Kaniewski N Schmaus A Niedhammer I Piciotti M Socioeconomic, demographic, occupational, and health factors associated with participation in a long-term epidemiologic survey: a prospective study of the French GAZEL cohort and its target population Am J Epidemiol 2001 154 4 373 384 10.1093/aje/154.4.373 11495861

[B23] Lindsted KD Fraser GE Steinkohl M Beeson WL Healthy volunteer effect in a cohort study: temporal resolution in the Adventist health study J Clin Epidemiol 1996 49 7 783 790 10.1016/0895-4356(96)00009-1 8691229

[B24] Zheng W Chow W-H Yang G Jin F Rothman N Blair A Li H-L Wen W Ji B-T Li Q The shanghai Women's health study: rationale, study design, and baseline characteristics Am J Epidemiol 2005 162 11 1123 1131 10.1093/aje/kwi322 16236996

[B25] Manolio TA Collins R Enhancing the feasibility of large cohort studies JAMA 2010 304 20 2290 2291 10.1001/jama.2010.1686 21098774 PMC3075846

[B26] Doll R Peto R Boreham J Sutherland I Mortality in relation to smoking: 50 years' observations on male British doctors BMJ 2004 328 7455 1519 10.1136/bmj.38142.554479.AE 15213107 PMC437139

[B27] Collaborative Group on Hormonal Factors in Breast Cancer Breast cancer and hormonal contraceptives: collaborative reanalysis of individual data on 53 297 with breast cancer and 100 239 women without breast cancer from 54 epidemiological studies Lancet 1996 347 1713 1727 8656904 10.1016/s0140-6736(96)90806-5

